# Intact myocardial preparations reveal intrinsic transmural heterogeneity in cardiac mechanics

**DOI:** 10.1016/j.yjmcc.2020.03.007

**Published:** 2020-04

**Authors:** Fotios G. Pitoulis, Waseem Hasan, Mary Papadaki, Nicolas G. Clavere, Filippo Perbellini, Sian E. Harding, Jonathan A. Kirk, Samuel Y. Boateng, Pieter P. de Tombe, Cesare M. Terracciano

**Affiliations:** a National Heart and Lung Institute, Imperial College London, UK; b Department of Cell and Molecular Physiology, Loyola University Chicago, USA; c School of Biological Sciences, University of Reading, UK; d Institute for Molecular and Translational Therapeutic Strategies, Hannover Medical School, DE, Germany

**Keywords:** Transmurality, Contractility, Cardiac mechanics, Intact tissue, Sarcomeric apparatus, Extracellular matrix

## Abstract

Determining transmural mechanical properties in the heart provides a foundation to understand physiological and pathophysiological cardiac mechanics. Although work on mechanical characterisation has begun in isolated cells and permeabilised samples, the mechanical profile of living individual cardiac layers has not been examined. Myocardial slices are 300 μm-thin sections of heart tissue with preserved cellular stoichiometry, extracellular matrix, and structural architecture. This allows for cardiac mechanics assays in the context of an intact in vitro organotypic preparation. In slices obtained from the subendocardium, midmyocardium and subepicardium of rats, a distinct pattern in transmural contractility is found that is different from that observed in other models. Slices from the epicardium and midmyocardium had a higher active tension and passive tension than the endocardium upon stretch. Differences in total myocyte area coverage, and aspect ratio between layers underlined the functional readouts, while no differences were found in total sarcomeric protein and phosphoprotein between layers. Such intrinsic heterogeneity may orchestrate the normal pumping of the heart in the presence of transmural strain and sarcomere length gradients in the in vivo heart.

## Highlights

• The myocardial slice preparation is an intact cardiac model allowing the study of transmural properties.
• Mechanical behaviour is cardiac layer dependent.
• Structural differences in cardiomyocyte density, orientation, and aspect ratio may contribute to these findings.

## Graphical abstract

**Figure:**
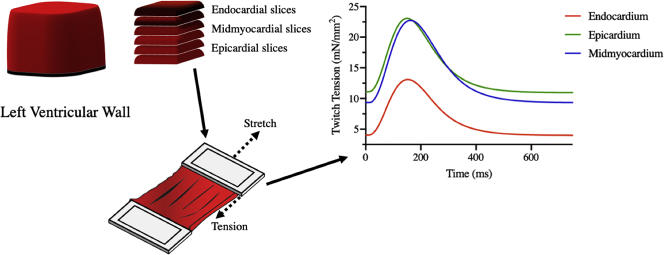

## 1 Introduction

The ventricular myocardium is increasingly recognized as a structure with regional variation. Although differences in electrical properties across the heart wall have been the subject of multiple studies, descriptions of transmural mechanical properties are marred by a paucity of data [1,2].

Determining mechanical behaviour across the heart wall is important as transmural differences in contractility can impact cardiac stroke volume, and changes in transmurality have been observed in human heart failure [2].

However, data on transmural mechanical behavior has been conflicting. Ambiguity remains with respect to whether the endocardium has a higher passive tension (i.e. is stiffer) [3,4] and active tension [3] than the epicardium or not [2,5,6]. Additionally, studies have been limited to isolated cardiomyocytes and permeabilised preparations. Such cardiac models provide insight into the cellular and subcellular basis of transmurality but are restricted in their ability to capture the properties of the in-situ myocardium. Isolated cardiomyocytes assess single cells independent of the effect of intercalated discs, extracellular matrix (ECM), multi- & hetero-cellularity in regulating contraction [7], and the transmural variation of these. Likewise, in permeabilised samples, sarcolemmal components and the ECM - both known to affect contractility - are disrupted. Therefore, whether transmural differences observed in these models translate to intact living tissue remains unanswered.

Myocardial slices are a cardiac model of intermediate complexity serving as a bridge between isolated cells and whole heart studies. Slices are 300 μm-thin living organotypic preparations with native cellular architecture, cell-cell/cell-ECM interactions, and preserved metabolic, electric, and mechanical properties [7]. Thus, slices are a novel intact physiological model on which to evaluate cardiac behavior. The slice model permits force vectors to be examined across a 2D plane, enabling mechanical insights comparable to those conducted on isolated cells and permeabilised preparations. Transmural cardiac mechanics were assessed in rat slices and the underlying structural, and sarcomeric differences explored.

## 2 Methods

### 2.1 Myocardial slice preparation

Myocardial slices were generated as described in [7]. Briefly, slices were prepared from the left ventricles of 11–15-week-old Sprague-Dawley rats (350–470 g) using a high-precision vibratome. Slices were generated in sequence, from endocardium to epicardium. Most ventricles yielded six slices. The earliest slice of 300 μm thickness, after removal of trabeculae carneae, was defined as the first slice. The 1st and 2nd slices were classified as subendocardial myocardium, the 3rd and 4th slice as midmyocardium, and the 5th and 6th slices as subepicardial myocardium. For clarity, these will be referred to as endocardium, midmyocardium, and epicardium.

### 2.2 Laser diffraction experiments

The % stretch-sarcomere length relationship was determined using laser diffraction. A high-powered laser was vertically directed on slices, which were progressively stretched, and the diffraction profiles processed in real-time (ImageJ, NIH, USA).

### 2.3 Contractility measurements

Contractility was assessed by mounting the slice on an isometric strain gauge (F30 Harvard Apparatus, UK), using custom 3D-printed holders glued on the slice. As myofiber direction rotates across the ventricular wall, holders were always attached perpendicular to main myofiber axis. All slices were stimulated at 1 Hz.

### 2.4 Sarcomeric protein content and phosphorylation status

Myofilament fractions were generated as previously described [8] from endo-, mid-, and epicardial slices. Total sarcomeric content and phosphoprotein status were quantified using Sypro Ruby and ProQ Diamond stain respectively.

### 2.5 Immunohistochemistry and picrosirius red staining

Cardiac slices were fixed and stained for caveolin-3, cardiac troponin T, and vimentin, and visualized under confocal microscopy within 1 day of staining. For collagen content hearts were cryosectioned, fixed, stained with Picrosirius red, and visualized under brightfield microscopy.

### 2.6 Data analysis

Contractility measurements were analysed using pClamp software (Molecular Devices, USA). Confocal and bright field images, as well as gels were analysed in ImageJ.

### 2.7 Statistical analysis

Data sets from each layer were analysed for statistical significance using ANCOVA and one-way ANOVA with Tukey's post-hoc test in Prism 8 (GraphPad, USA). *P* < .05 was considered statistically significant.

A detailed description of the methodology is available in the online supplement.

## 3 Results and discussion

**Fig. 1:**
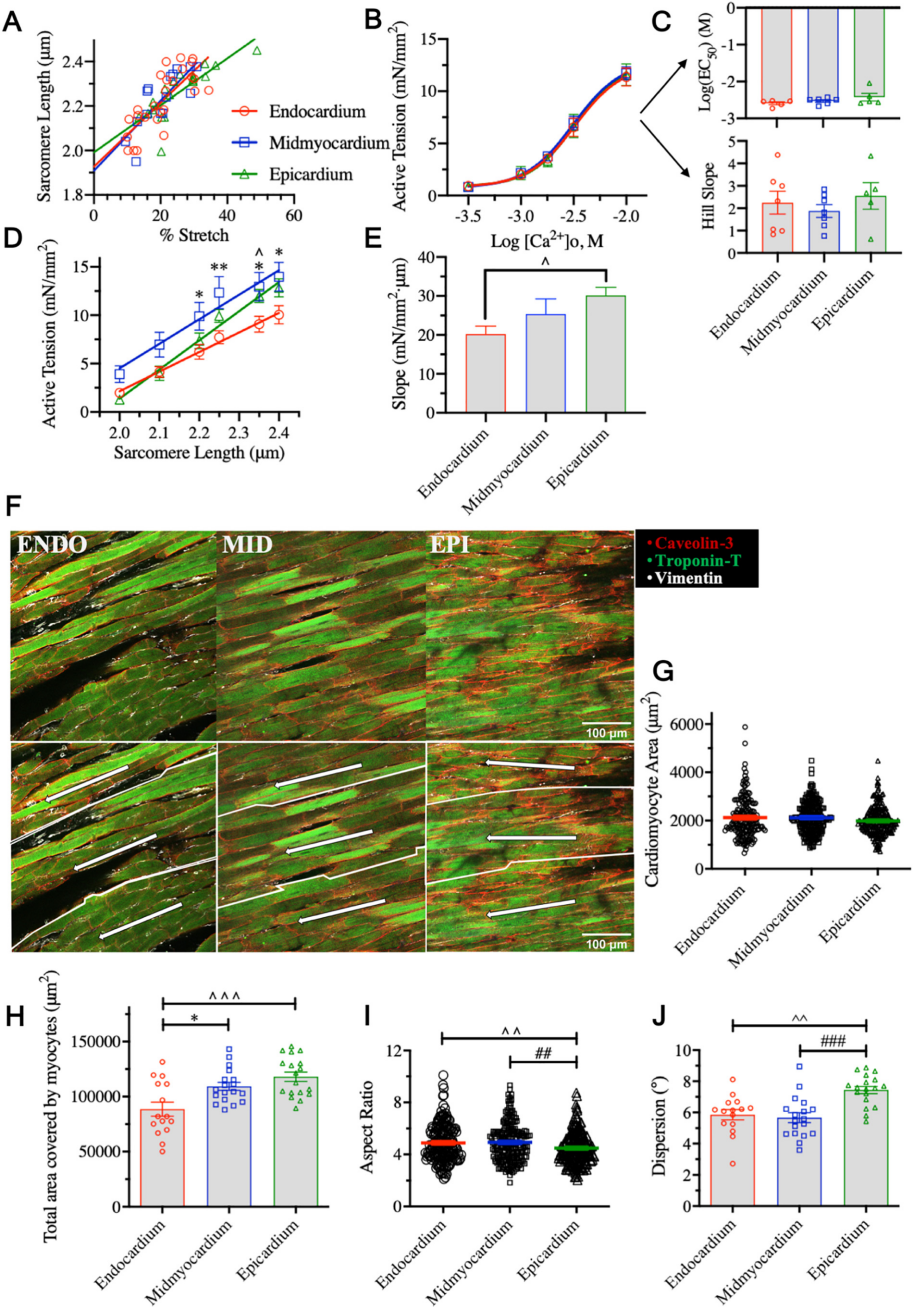
A) Transmural strain-sarcomere length (SL) relationship [*N* = 6]. B) Concentration-force response curves of cardiac slices from different layers to the external Ca^2+^ concentration ([Ca^2+^]o) [*N* = 8, *N* = 7, N = 6]. All force-Ca^2+^ experiments were conducted at 2.1 μm SL. C-Top) Transmural calcium sensitivity as measured by the log(EC_50_) [*N* = 5, N = 6, N = 5]. The variable slope concentration-response model fitted did not yield an EC_50_ for three endocardial, one midmyocardial, and one epicardial data sets. C-Bottom) Hill coefficients of the concentration-response curves of each cardiac layer [*N* = 7, N = 7, *N* = 5]. The variable slope concentration-response model fitted yielded an ambiguous slope for one endocardial, and one epicardial data set. D) Active tension-SL relationship for cardiac slices from different layers of the wall [N = 7, N = 5, N = 7]. E) Slopes of linear regression lines fit to the active tension-SL relationship [N = 7, N = 5, N = 7]. F) Representative confocal images of cardiomyocyte area, density, morphology, and direction. Each image in the bottom panel has been separated into three regions and the major direction of cardiomyocyte orientation drawn with an arrow. The greater the angle between the arrows the greater the variability in cardiomyocyte orientation. G) Cardiomyocyte area [*N* = 180/5, *N* = 216/6, N = 216/6]. H) Total myocyte area coverage (myocyte area × number of cardiomyocytes) [*N* = 15/5, N = 18/6, N = 18/6]. I) Cardiomyocyte aspect ratio [N = 180/5, N = 216/6, N = 216/6]. J) Cardiomyocyte dispersion [N = 15/5, N = 18/6, N = 18/6]. All analysis was done blinded. *: midmyocardium vs. endocardium, ^: epicardium vs. endocardium, #: midmyocardium vs. epicardium. [N = endocardium, midmyocardium, endocardium, and N = images/biological replicates].

Cardiac layers have been suggested to operate at different sarcomere lengths (SL) in vivo [3,9]. We identified the relationship between % stretch from slack length and SL change (Fig. 1A) using laser diffraction. This allowed control of SL, so that intrinsic differences of each cardiac layers to the same strain could be delineated.

As force production is a function of external Ca^2+^ concentration ([Ca^2+^]o), different force sensitivity to [Ca^2+^]o would mask intrinsic contractile differences. To account for this, we conducted a series of force-[Ca^2+^]o experiments with slices stretched to 2.1 μm SL (Fig. 1B). Ca^2+^ sensitivity, defined as the [Ca^2+^]o at which half-maximum force is generated (EC_50_), was not significantly different between layers (Fig. 1C-Top). It is worth noting that this EC_50_ is a different parameter to the one reported in permeabilised preparations, which describes the response of intracellular components to Ca^2+^. Permeabilised human endocardial preparations for example, exhibit greater Ca^2+^ sensitivity than the epicardium [2]. In contrast, our data suggests that the Ca^2+^ sensitivity of the intact tissue is homogeneous across the wall. Moreover, the Hill slope, which measures the steepness of the force-[Ca^2+^]o curve, was not different between layers, further supporting a uniform contractile response to Ca^2+^ (Fig. 1C-Bottom). These experiments yielded a known Ca^2+^ concentration (EC_50_ = 10^–2.54^ M) at which the mechanical properties of the heart could be reliably assessed while controlling for [Ca^2+^]o.

To determine contractile profiles across the wall, Frank-Starling experiments were performed at a range of SLs (2.00–2.40 μm). The midmyocardium produced a significantly higher active tension than the endocardium at SLs 2.20–2.40 μm (*p* < .05 and *p* < .01). Higher isometric force and power output has similarly been reported in permeabilised midmyocardium [2]. The epicardium also trended towards higher force development compared to the endocardium (Fig. 1D, p < .05 at SL of 2.35 μm). Contrasting data exists regarding active tension between these latter two layers and results appear to be species-dependent; in ferrets, the endocardium is reported to have a higher maximum active tension than the epicardium [3] whereas in pigs [6] and, more comparably, in rats no differences are described [3]. To quantify the ability of each layer to increase its force output upon stretch, a linear regression was fit to the active tension-SL relationship (Fig. 1D). The slope of this was significantly higher in the epicardium compared to endocardium (Fig. 1E, *p* < .05), suggesting greater contractile output per μm of stretch. When visualized under light microscopy, slices from the endocardium appear patchier and with recurring gaps between myofibers compared to other layers. Thus, we hypothesized that differences in the cellular composition and tissue architecture may exist and play a role in shaping the observed transmural mechanical heterogeneity.

Cardiomyocyte area, which has been reported to be higher in isolated rat endocardial than epicardial myocytes [10], was quantified (Fig. 1F-G)*.* Although no differences were found in individual myocyte area, total myocyte coverage of the tissue (myocyte area × number of myocytes) was significantly higher in both midmyocardium and epicardium compared to endocardium (*p* < .05, and *p* < .001 respectively) (Fig. 1H). Cardiomyocyte area is a major determinant of systolic force production and cardiomyocyte density correlates positively with force production in engineered heart tissue [11]. An absolute greater myocyte coverage would thus explain the increased active tension-SL relationship and steeper force-stretch response of the midmyocardium and epicardium. However, cardiomyocyte morphology can also impact cardiac contraction with long and thin myocytes at a mechanical disadvantage when generating force compared to shorter and thicker cells [12]. The cardiomyocyte aspect ratio (length:width) was significantly lower in epicardial cells compared to both midmyocardium and endocardium (Fig. 1I, *p* < .01).

Despite the lower aspect ratio, the force-stretch experiments showed that midmyocardium produces a higher active tension compared to the endocardium more consistently than the epicardium, and does so at a lower SL. We hypothesized that cardiomyocyte orientation could account for this. Cardiomyocyte dispersion, a measure of the standard deviation of a Gaussian curve fitted to the different angulations of structures (i.e. cardiomyocytes) in an image from the main axis of direction was quantified. Dispersion was significantly higher in the epicardium compared to endocardium and midmyocardium, suggesting greater cardiomyocyte orientation variability (Fig. 1J, *p* < .01 and *p* < .001 respectively). As myocyte misalignment can reduce force development [13], higher cardiomyocyte disorientation may offset force production in the epicardium despite the lower aspect ratio and explain why the midmyocardium tends to develop marginally higher active tension than the epicardium when compared to the endocardium (Fig. 1D-E).

**Fig. 2:**
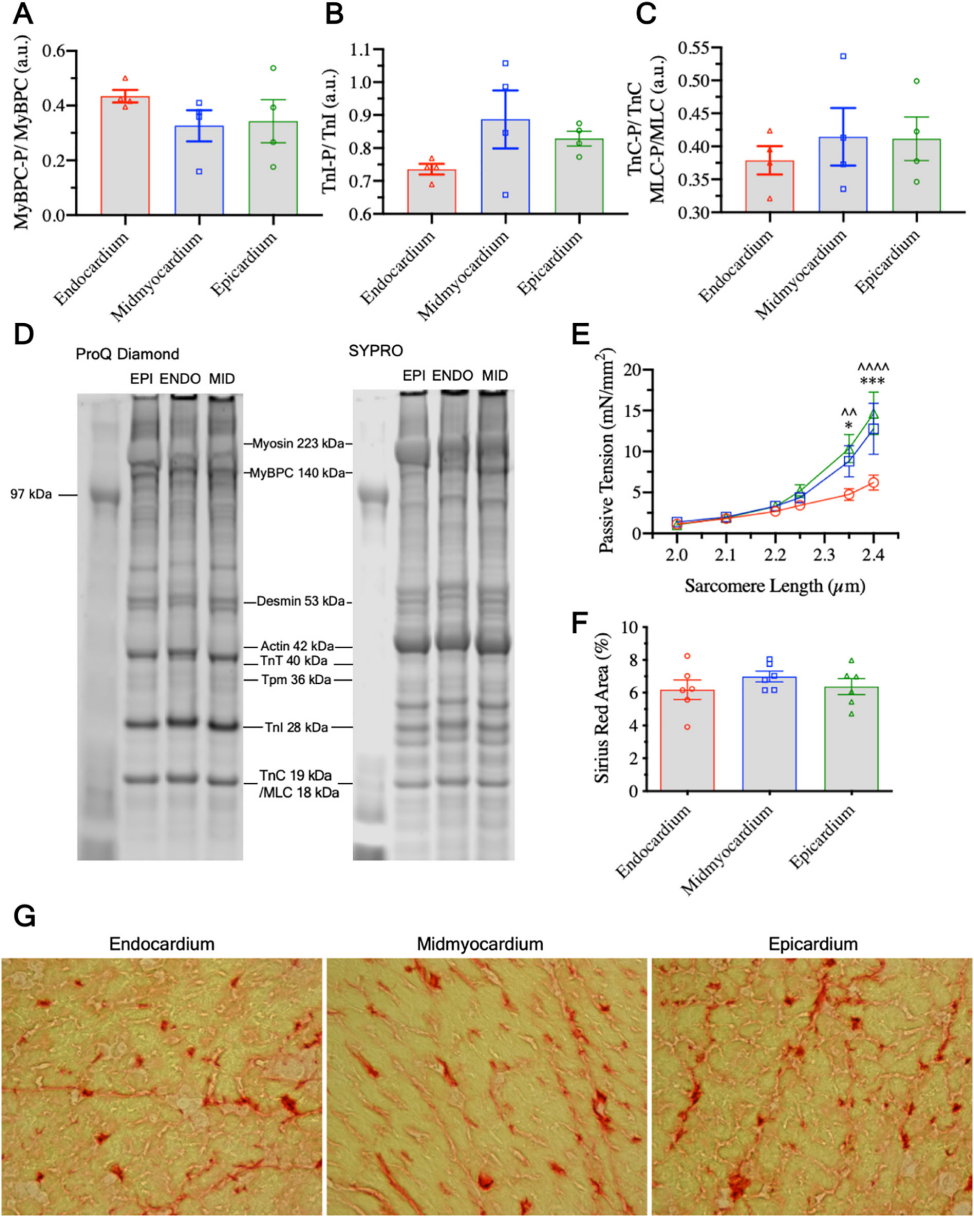
A-C) Ratio of phosphorylated myosin binding protein C, troponin I, and troponin C & myosin light chain to unphosphorylated counterparts respectively [*N* = 4]. D) Bands from total sarcomeric content (right) and phosphoproteins (left) separated by molecular weight. E) Passive tension-SL relationship for cardiac slices from different layers of the wall [*N* = 7, *N* = 5, N = 7]. F) % Area covered by Sirius red staining from transverse cryosections [*N* = 6]. G) Representative brightfield images of Sirius red staining.*: midmyocardium vs. endocardium, ^: epicardium vs. endocardium. [N = endocardium, midmyocardium, endocardium]. (For interpretation of the references to colour in this figure legend, the reader is referred to the web version of this article.)

The sarcomeric apparatus could also underlie transmural differences in active tension. As such, we performed total sarcomeric protein content and phosphoprotein status quantification using Sypro Ruby and ProQ Diamond staining. However, our results showed no significant differences across the wall despite a gradient tendency of certain proteins (Fig. 2A-D). Comparable transmural uniformity of sarcomeric proteins has been reported in the non-failing human heart [2] and rats of similar age by others [14].

Passive tension was significantly lower in the endocardium compared to midmyocardium and epicardium at 2.35 μm and 2.40 μm SL (Fig. 2E, *p* < .001 and *p* < .0001 at SL of 2.40 μm). In intact isolated cardiomyocytes, passive tension increases more in the endocardium than epicardium with SL [3]. Data from skinned samples similarly show a stiffer endocardium at high SLs [4], although others have reported no differences between the two [2,5] nor between the midmyocardium and the other layers [2]. Our slice data show that intact endocardial layers are less stiff.

Passive tension is particularly important during the refilling phase of the cardiac cycle. Reconstruction of in vivo transmural SL gradients using radiopaque beads and biplane cineradiography demonstrate that the endocardium operates at a higher and narrower SL range than the mid- and epicardium, that can reach 2.4 μm length, where our significant differences arise, prior to ejection [9]. Likewise, the loading cycle of the isolated arrested heart shows clear transmural deformations (i.e. normal strains – longitudinal, radial, and circumferential – increase from epicardium to endocardium) [15]. The consequences of a) transmural strain gradients, but b) uniform SL-stretch relationships (Fig. 1A) in the presence of a more compliant endocardium (Fig. 2E) is a greater deformation of the inner myocardium enabling it to attain a higher SL. Under this conceptual framework, lower compliance of outer layers would similarly facilitate their operation at lower SLs while averting excessive endocardial diastolic strain, in effect acting as ‘guardian’ layers.

These conclusions are important not only for diastole but also active cardiac contraction. Transmurally non-uniform SLs would position the endocardium further up and to the right of the Frank-Starling curve and the outer layers further down and to the left. Intrinsic differences in contractility (Fig. 1A-B), largely attributed here to structural heterogeneity and total myocyte coverage (Fig. 1G-J), may thus be offset, homogenizing force production and ventricular pumping efficiency [1]. In support of this, cardiomyocytes with low aspect ratio develop the highest systolic work in stiff environments whereas those with higher aspect ratio fare better in less stiff environments [16], which is in agreement with our functional and structural data.

To uncover the structural factors involved in differential passive tension we performed transverse sectioning of tissue followed by picrosirius red staining. We found no significant differences in total collagen content between layers (Fig. 2F-G). Although collagen is a known determinant of passive myocardial mechanics, stiffness is dominated by titin content and isoform composition. In the adult rat, titin, a giant molecular sarcomeric protein, exists either as larger compliant N2BA or smaller stiffer N2B isoform. Although we were unable to quantify this, others have reported extensively that in healthy canine and pig hearts, the N2BA:N2B ratio shows a transmural gradient highest in the subendocardium [17], consistent with our mechanical data. Transmural heterogeneity in cross-bridge cycling could also underlie the observed differences. Testing this aspect would be insightful and will be part of future investigations.

A number of limitations are discussed. First, myocardial slices generate stress and respond to strain across a 2D-plane. This is in contrast to the in vivo 3D operation of the heart, where pressure is generated corresponded with distinct changes in volume. Additionally, fiber alignment is known to rotate across the 3D ventricular wall, in contrast to the uniaxial plain of examination in slices. As slices from adjacent layers (1st and 2nd slice & 5th and 6th slice) were collapsed together for data analysis, transmural differences may have been underestimated. Another limitation is the use of muscle length control for isometric measurements, which may introduce experimental error due to damaged end-compliance [18]. However, the tension-SL relationship with muscle length has been suggested to be the same as that of SL control [19]. Our data supports that functional differences in transmural mechanics are dominated by structural heterogeneity and not sarcomeric protein content or phosphorylation status. Transmural gradients in myosin light chain phosphorylation have been reported [20] and suggested to explain the pattern of active mechanical contraction, which is dominated by the outer layers. However, our results like those of others did not show the presence of such spatial gradients [21].

In conclusion, we show for the first time that in intact tissue, intrinsic differences exist in myocardial active and passive mechanics that are primarily governed by structural transmural heterogeneity. In vivo, operation of cardiac layers at different SLs has been shown and our findings provide a physiological explanation for this; differences in operating SL seem to be balanced out by differences in the intrinsic mechanical properties.

## Disclosures

Part of this study was featured as an abstract in the European Society of Cardiology Congress, Paris 2019. All authors declare no conflicts of interest, financial or otherwise.

## Appendix A Supplementary data

Supplementary material Image 1
