# Supplementary material for: Intact myocardial preparations reveal intrinsic transmural heterogeneity in cardiac mechanics
Source: J Mol Cell Cardiol. 2020 Apr;141:11–6. doi: 10.1016/j.yjmcc.2020.03.007 (PMC7246333; doi:10.1016/j.yjmcc.2020.03.007)
Supplement: Supplementary file 1 — Supplementary material [file mmc1.docx]

## Supplementary Material

# 4.1. Myocardial Slice Preparation

All animal experiments were conducted in accordance with institutional and national regulations and were approved by Imperial College London, under license by the UK Home Office, United Kingdom Animals (Scientific Procedures) Act 1986.

Myocardial slices were prepared using the protocol previously detailed by Watson et al.[7]. Adult Sprague-Dawley rats (aged 11-15 weeks and 370-450g weight) were sacrificed under anaesthesia (4% isoflurane at 4 L/min oxygen), their heart explanted and placed in 50 ml of heparinized (1000IU/ml) modified Tyrode solution (30.0 mM 2, 3-Butanedione Monoxime, 140.0 mM NaCl, 9.0 mM KCl, 10 mM Glucose, 10.0 mM HEPES, 1.0 mM Magnesium Chloride, 1.0 mM Calcium Chloride; pH 7.40) at 37°C and shortly thereafter at 4°C heparinized modified Tyrode solution. The left ventricle was isolated by removing the lungs and other extra-cardiac structures using a surgical blade and dissecting away the atria and right ventricle. It was then opened up, flattened and the epicardial side glued (Histoacryl®, Braun, Germany) onto a layer of agarose (4% agar) on a specimen holder. The specimen holder was mounted to the tissue bath of a high precision vibrating microtome (7000 smz2, Campden Instruments Ltd., UK) previously filled with oxygenated 4°C modified Tyrode solution.

Before slicing, the vibratome was fitted with a ceramic blade, calibrated to a Z-axis error <1.0 μm and the blade adjusted to have an amplitude of 2 mm, vibration frequency of 80 Hz and advance speed of 0.03 mm/s. These settings were chosen to produce highly viable 300 μm myocardial slices. Each slice generated was examined under light microscopy to determine myocardial fiber orientation. One section with parallel fiber orientation and from the mid-region of each slice was selected and cut into a rectangular sample. The latter was important to minimize apico-basal differences which could confound the results. The length and width of the slice were measured using calipers and recorded for normalization of force to the slice cross-sectional area. For slices used in the contractility, laser diffraction experiments as well as for fixation, custom-designed 3D-printed Polylactic acid (PLA) holders were attached to both ends of the sample perpendicular to the fiber orientation using surgical glue. These were used to manipulate and mount the slice to the force-transducer, laser diffraction, or tissue fixation set-ups.

# 4.2. Laser Diffraction Experiments

Myocardial slices were mounted on custom-made stainless-steel stretcher using the 3D printed holders. The stretcher was positioned into a glass dish, filled with modified Tyrode’s solution at room temperature. A high powered HeNe laser (Lasos, Germany) was positioned 2 cm vertically above the slice. The laser was turned on and as it passed through the slice, it diffracted into bands based on the amount of tissue stretch. A camera (Logitech C920 HD) was used to analyze the diffraction profiles in real-time using the plot-profile functionality of ImageJ (National Institute of Health, USA). Each slice was progressively stretched (from slack length) to three different muscle lengths, and the data were used to determine the % stretch-sarcomere length (SL) relationship. To account for SL inhomogeneity across the tissue, the laser was directed at three different regions of the myocardial slice at each muscle length, the SL determined, and then averaged for each stretch. A linear regression model was fit to the % stretch-SL relationship for slices from each cardiac layer. Given a known slice slack length and the linear relationship between stretch and SL, the muscle length correspondent with a desired SL could be calculated. This was subsequently used for fine manipulation of strain in force-calcium and Frank-Starling experiments.

4.3. Force-Calcium Experiments

Normal Tyrode’s solutions (140.0 mM NaCl, 4.5 mM KCl, 10 mM Glucose, 10.0 mM HEPES, 1.0 mM Magnesium Chloride; pH 7.40) with calcium concentrations of 0.316 mM (10^-3.5^ M), 1 mM (10^-3^ M), 1.8 mM (10^-2.7^ M), 3.16 mM (10^-2.5^ M), 10 mM (10^-2.0^ M) were prepared.

Myocardial slices were moved into an organ bath chamber and mounted on an isometric strain gauge (F30 Harvard Apparatus, USA) using the PLA holders. The organ bath contained oxygenated 1.8 mM Tyrode’s solution at 25°C (starting temperature). Electrical stimulation was initiated, and the temperature of the solution slowly raised until 36 ± 0.5°C. Slices were stretched to a SL of 2.1 μm using muscle length as a surrogate. A micromanipulator and video camera connected to and calibrated with the ImageJ software (National Institute of Health, USA) webcam plugin were used for real-time measurements of muscle-length. Slices were stimulated at 1 Hz, 20-30V amplitude and 10-20 ms width biphasic pulses and data was acquired using AxoScope software (Molecular Devices, USA). After one minute of steady state force production was achieved, perfusion of the current solution was replaced with a randomly chosen Tyrode’s solution containing an unused calcium concentration. This process was repeated until measurements from all calcium concentrations were obtained. At the end, slices were again assessed at 1.8 mM Tyrode’s solution and if the active tension generated was <60% of the active tension at the first 1.8mM solution, slices were excluded from further analysis.

The pClamp software package (Molecular Devices, USA) was used to analyze three force transients generated at the steady state of each calcium concentration (force-calcium) and SL (Frank-Starling – see section 4.4.). The forces generated by the slices were normalized to their cross-sectional area (slice thickness 0.3 mm $x$ slice width). Active tension generated during the force-calcium experiments were plotted against a log scale of Ca^2+^ concentration and a sigmoidal log(agonist) vs. response (variable slope, four parameters) model was fitted according to the equation:

$$A=B+C\frac{1}{1+{10}^{(({logEc}_{50}-\log{Ca}^{2+}))*H}}$$

where A is the active tension, B is the passive tension, C is the maximum force generated, H is the hill coefficient and EC_50_ is the calcium concentration required to reach half maximum active tension.

4.4. Frank-Starling Experiments

Myocardial slices were mounted onto the isometric strain gauge in the organ bath, containing oxygenated 25°C Tyrode’s solution but with 10^-2.54^ M Ca^2+^ (EC_50_) and slowly heated to 36 ± 0.5°C. The slices were progressively stretched to muscle lengths correspondent to SL of 2.0-, 2.10-, 2.20-, 2.25-, 2.35- and 2.40 μm. Slices from different layers were stretched to each SL based on their individual SL-% stretch profiles determined previously from the laser diffraction experiments. All slices were field stimulated at 1 Hz, 20-30V amplitude and 10-20 ms width biphasic pulses using platinum electrodes.

A linear regression was fit to the active tension-SL relationship; the gradient of each line was compared to determine the force sensitivity of each cardiac layer to stretch. R^2^ values were 0.72, 0.60, and 0.84 for endocardium, midmyocardium, and epicardium respectively.


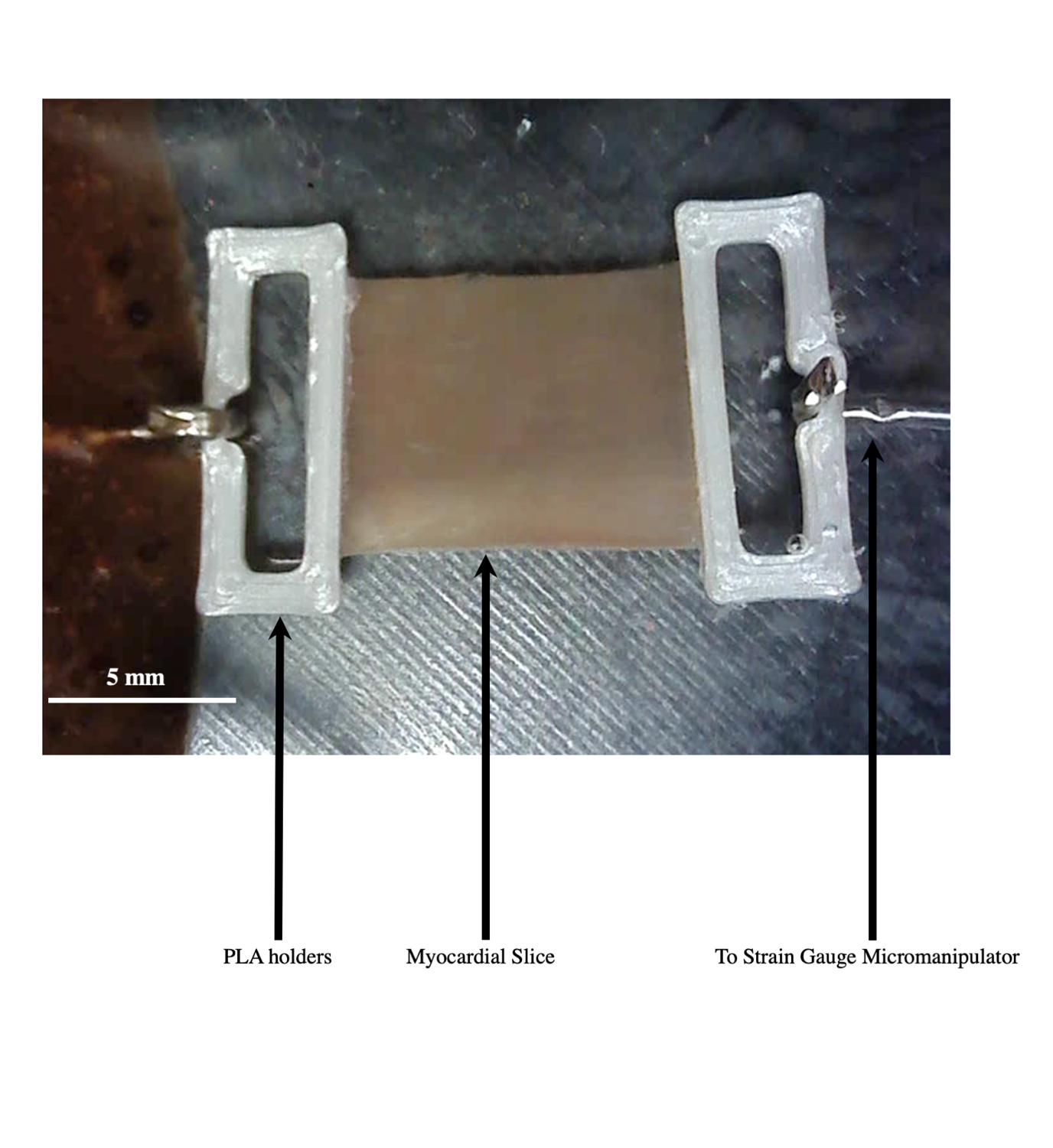


**Figure 1 Supplementary:** Myocardial slice inside the organ bath. 3D-printed PLA holders are attached perpendicular to the direction of myocardial fibers and used to mount the slice on an isometric strain gauge. The slice is stimulated with platinum electrodes (not visible here) at 1Hz and the active and passive tension recorded.

## 4.5. Immunohistochemical Staining for Confocal Imaging

Cardiac slices were mounted on stretchers, stretched to 2.1 μm SL, and fixed in 4% formaldehyde for 15 minutes at room temperature, washed in phosphate buffered solution (PBS, Sigma), permeabilised and blocked using 1.5% Triton X-100 in 10% FBS, 5% BSA and 10% horse serum for three hours at room temperature on a rocker. Slices were then washed in PBS three times and incubated with their primary antibodies overnight at room temperature on a rocker. After being washed a further three times in PBS, they were incubated with their secondary antibody for 2 hours at room temperature on a rocker, covered with aluminum foil. After a final three washes, the samples were stored at 4ºC in PBS. All stained slices were visualized under confocal microscopy within 1 day of being stained. Myocardial slices were viewed under a confocal microscope (Zeiss LSM-780) and Z-stack images from three different regions were taken from each slice at 20x magnification.

Confocal images were analyzed blinded in ImageJ. Caveolin-3 stained the membrane of the cardiomyocytes and allowed identification of the cells and their borders. Length of the cell was defined as the longest axis across a cardiomyocyte and width as the longest line perpendicular to the length axis. Cell area was measured by circumscribing along the cardiomyocyte borders demarked by caveolin-3 staining. Cell density was quantified by counting the number of fully visible cells present in each image. 12 cells were analysed for length:width ratio, and area from each image. Total myocyte area was determined as the product of number of cardiomyocytes × cardiomyocyte area. Vimentin levels were obtained from each image’s first four Z-stacks and were normalized using the auto-threshold settings on ImageJ. They were subsequently measured using the pixel quantification tool. Dispersion was calculated using data from all Z-stacks available in the image and measured with the software’s directionality analysis tool.

**Table 1:** Antibodies used to stain cardiac slices

| **Antibody** | **How it is created** | **Dilution** |
| --- | --- | --- |
| **Primary Antibodies** | | |
| Caveolin 3 | Mouse | 1:500 |
| Cardiac Troponin T | Rabbit | 1:800 |
| Vimentin | Chicken | 1:3000 |
| **Secondary Antibodies** |  |  |
| Alexa Fluor 488 | Donkey, anti-rabbit | 1:2000 |
| Alexa Fluor 546 | Donkey, anti-mouse | 1:2000 |
| Alexa Fluor 647 | Goat, anti-chicken | 1:2000 |

4.6. Sarcomeric Proteins

Myofilament fractions were prepared from endocardium, midmyocardium, and epicardium as previously described[8]. Myofilament proteins (20 μg per lane) were run on a 4-12 % gradient gel. The gel was then stained with ProQ Diamond according to manufacturer’s instructions (Invitrogen). Briefly, the gel was fixed in a 10% acetic acid, 50% methanol solution for one hour. It was then stained with ProQ stain for 75 mins and destained with a solution containing 20% acetonitrile, 50 mM sodium acetate, pH 4.00. The resulting gel was imaged using the Typhoon scanner (GE Healthcare) at 580 nm. After imaging, it was stained overnight with Sypro Ruby stain (Invitrogen) and after destain with 10% methanol, 7% acetic acid, it was imaged using the Typhoon scanner at 619 nm. Band quantification was performed using Image J and the ratio of each phosphorylated protein to total protein was calculated.

4.7. Picrosirius Red Staining

The harvested hearts of six SD rats were blocked in Optimal Cutting Temperature media and stored at -80°C for 24 hours. 7 μm transverse sections were taken by cutting the OCT blocks with a cryostat at -23°C and harvested on slides, before being stored at -80°C. To visualize cardiac fibrosis, sections were stained with Picrosirius Red staining kit (ab150681, Abcam). To do that, they were prefixed in Bouin solution for 15 minutes under fume hood at room temperature then washed for 15 minutes at room temperature in tap water. Then, sections were stained in Picrosirius red for 1 hour at room temperature under the fume hood. Slides were differentiated in acidified water, twice, two dips. Sections were then washed and dehydrated three times in 100% Ethanol before being cleared in Xylene over five minutes. Imaging was done with brightfield microscope Nikon TE200. 10 pictures per area of interest per section were obtained, and two sections were analysed for each animal. The quantification of the red stain was carried out with ImageJ. In a first time the original picture was changed to RGB format. Then, a threshold previously calibrated in order to cover most of the staining was applied on the RGB picture and the area covered by the stain measured.

## 4.8. Statistical Analysis

For comparison of statistical significance between linear regression lines (laser diffraction and active tension-SL relationships) analysis of co-variance (ANCOVA) was used. For contractility (active-tension, passive-tension, logEC_50_, Hill slope) and confocal images, the data sets of slices from each layer were compared using one way analysis of variance (ANOVA) with Tukey‘s post-hoc in Prism8 software (GraphPad, USA). P<0.05 was considered statistically significant**.**
